# Individualized dosing in the precision medicine era: a narrative review

**DOI:** 10.3389/fphar.2026.1944686

**Published:** 2026-10-07

**Authors:** Muath A. Alsalloum, Jude Howaidi, Faris S. Alnezary, Masaad S. Almutairi, Anwar A. Alsakaker

**Affiliations:** 1 Department of Pharmacy Practice, College of Pharmacy, Qassim University, Buraydah Qassim, Saudi Arabia; 2 Clinical Pharmacy Department, King Fahad Medical City, Riyadh, Saudi Arabia; 3 Department of Pharmacy Practice, College of Pharmacy, Taibah University, Madinah, Saudi Arabia; 4 College of Pharmacy, Qassim University, Buraydah Qassim, Saudi Arabia

**Keywords:** fixed dosing, individualized dosing, pharmacokinetics/pharmacodynamics, precision dosing, therapeutic index, personalized medicine

## Abstract

Individualized dosing has become a cornerstone of precision medicine by optimizing therapeutic efficacy while minimizing toxicity through patient-specific dose selection. Traditional approaches, including weight- and body surface area–based dosing, remain fundamental for many medications but are increasingly complemented by pharmacokinetic/pharmacodynamic (PK/PD)-guided strategies and model-informed precision dosing (MIPD). This narrative review summarizes current evidence on individualized dosing across four major therapeutic classes: antimicrobial agents, chemotherapy, monoclonal antibodies and immunotherapies, and anesthetic and critical care agents. It highlights the rationale, advantages, and limitations of conventional dosing approaches while discussing the growing adoption of fixed dosing for selected biologics with predictable pharmacokinetics. The review also examines practical challenges associated with individualized dosing, including dose calculation complexity, medication errors, and operational constraints. Finally, emerging precision-dosing strategies, particularly Bayesian adaptive dosing and biomarker-guided dosing, are discussed as promising approaches to further personalize pharmacotherapy. Collectively, these advances support a shift toward more precise, evidence-based, and patient-centered medication dosing.

## 1 Introduction

Flat dosing is a dosing strategy in which a fixed, uniform dose of a medication is given to all patients, irrespective of patient-specific variables such as body weight, body surface area (BSA), sex, or organ function. While flat dosing is characterized by simplicity and a reduced risk of calculation errors, individualized/weight-based dosing offers greater precision by minimizing the likelihood of under- or over-dosing and reducing the potential for adverse effects (Tyson et al., 2020; Pan et al., 2016; Pai, 2012).

Small, lipophilic agents, renally eliminated drugs, and medications with a narrow therapeutic index (TI) are associated with an increased risk of concentration-dependent toxicity and are therefore commonly administered using a weight-based dosing strategy (Pan et al., 2016; Pai, 2012; Hendrikx et al., 2017). From a pharmacokinetic perspective, small, lipophilic molecules often exhibit tissue distribution and variable volume of distribution (Vd), which may correlate with body mass and composition (Pan et al., 2016; Benet, 2018). Fixed dosing in such cases can result in substantial interindividual variability in systemic exposure. Similarly, renally cleared drugs demonstrate clearance (Cl) that is closely linked to renal function, which may correlate with body size and glomerular filtration rate (GFR) (Pan et al., 2016; Prager et al., 2025). Without dose individualization, patients at extremes of body weight may experience supratherapeutic or subtherapeutic plasma concentrations (Pan et al., 2016; Pai, 2012). Additionally, Medications with a narrow TI are particularly sensitive to even modest deviations in systemic exposure, as the therapeutic and toxic concentration ranges are closely aligned (Derendorf and Meibohm, 1999). For these agents, weight-based dosing (e.g., mg/kg) can reduce variability in drug exposure, mitigate the risk of overdosing, and improve the probability of maintaining concentrations within the therapeutic window (Tyson et al., 2020; Pai, 2012). Collectively, these pharmacokinetic and pharmacodynamic (PD) considerations provide a mechanistic rationale for preferring weight-based dosing in clinical scenarios where interindividual variability meaningfully influences drug safety and efficacy (Tyson et al., 2020).

The TI plays a central role in determining the need for dosing individualization, as it reflects the margin of safety between therapeutically effective and toxic drug exposures (Derendorf and Meibohm, 1999). The TI is traditionally defined as the ratio between a toxic dose (e.g., TD_50_) and an effective dose (e.g., ED_50_) (Derendorf and Meibohm, 1999; Brunton et al., 2018). Clinically, however, it is more relevant to consider the therapeutic window, which represents the plasma concentration range within which a drug is both effective and safe (Derendorf and Meibohm, 1999). A substantial separation between effective and toxic concentrations is associated with a wide TI, whereas a minimal separation between the two is associated with a narrow TI (Derendorf and Meibohm, 1999; Brunton et al., 2018). Therefore, the narrower the TI, the greater the clinical imperative for dosing individualization (Tyson et al., 2020; Derendorf and Meibohm, 1999).

Individualized dosing refers broadly to the selection or adjustment of a drug regimen according to patient-specific characteristics or treatment-response information with the aim of optimizing efficacy and minimizing toxicity. Precision dosing represents an approach within precision medicine that uses patient-specific information to optimize drug therapy and may incorporate pharmacokinetic, clinical, and biological information to account for interindividual variability in drug exposure or response. Importantly, the approaches discussed in this review are related but not interchangeable and should not be considered a strictly sequential continuum. Weight- or body surface area–based dosing represents a relatively simple form of dose individualization in which measurable patient characteristics are used as dosing covariates. Therapeutic drug monitoring (TDM) individualizes therapy using measured drug concentrations to assess exposure and guide subsequent dose adjustment. Model-informed precision dosing (MIPD) uses pharmacokinetic or PK/PD models, often incorporating patient-specific covariates and drug concentrations, to estimate individual pharmacokinetic parameters and optimize dosing; Bayesian adaptive dosing is one important implementation of MIPD in which individual observations are combined with prior population information to update patient-specific estimates. Biomarker-guided dosing, in contrast, uses measurable biological signals reflecting drug effect, disease activity, or physiological response to inform treatment adjustment and may be applied independently or in combination with pharmacokinetic approaches. Although precision medicine encompasses broader approaches, including pharmacogenomics and other molecularly informed strategies, this review focuses specifically on individualized drug dosing and its practical implementation. Pharmacogenomic approaches are acknowledged where relevant but are not comprehensively reviewed. Thus, these strategies represent complementary tools for individualizing therapy, with their applicability determined by the drug, therapeutic target, patient population, available measurements, and clinical context.

Figure 1 illustrates individualized dosing as a component of precision medicine and the complementary approaches used to optimize drug therapy based on patient-specific information. This narrative review aims to summarize the scientific rationale for individualized dosing strategies in contemporary clinical practice and to examine their application across major therapeutic areas, including antimicrobials, chemotherapy, biologics and immunotherapies, and anesthetic and critical care agents. These four therapeutic classes were selected because they encompass diverse clinical scenarios and dosing paradigms in which individualized dosing has particular relevance. Antimicrobial agents illustrate PK/PD-driven dosing and therapeutic drug monitoring; chemotherapy represents traditional body surface area–based dosing and drug-specific dose individualization; monoclonal antibodies and immunotherapies highlight the transition from weight-based to fixed dosing for selected agents; and anesthetic and critical care agents demonstrate the importance of individualized dosing in settings characterized by rapid titration and dynamic physiological changes. Collectively, these classes provide a broad framework for examining the principles, challenges, and emerging approaches to individualized dosing across contemporary pharmacotherapy. In addition, it discusses the practical challenges associated with individualized dosing and examines complementary approaches to dose individualization, including therapeutic drug monitoring, model-informed precision dosing, Bayesian adaptive dosing, and biomarker-guided dosing.

**FIGURE 1:**
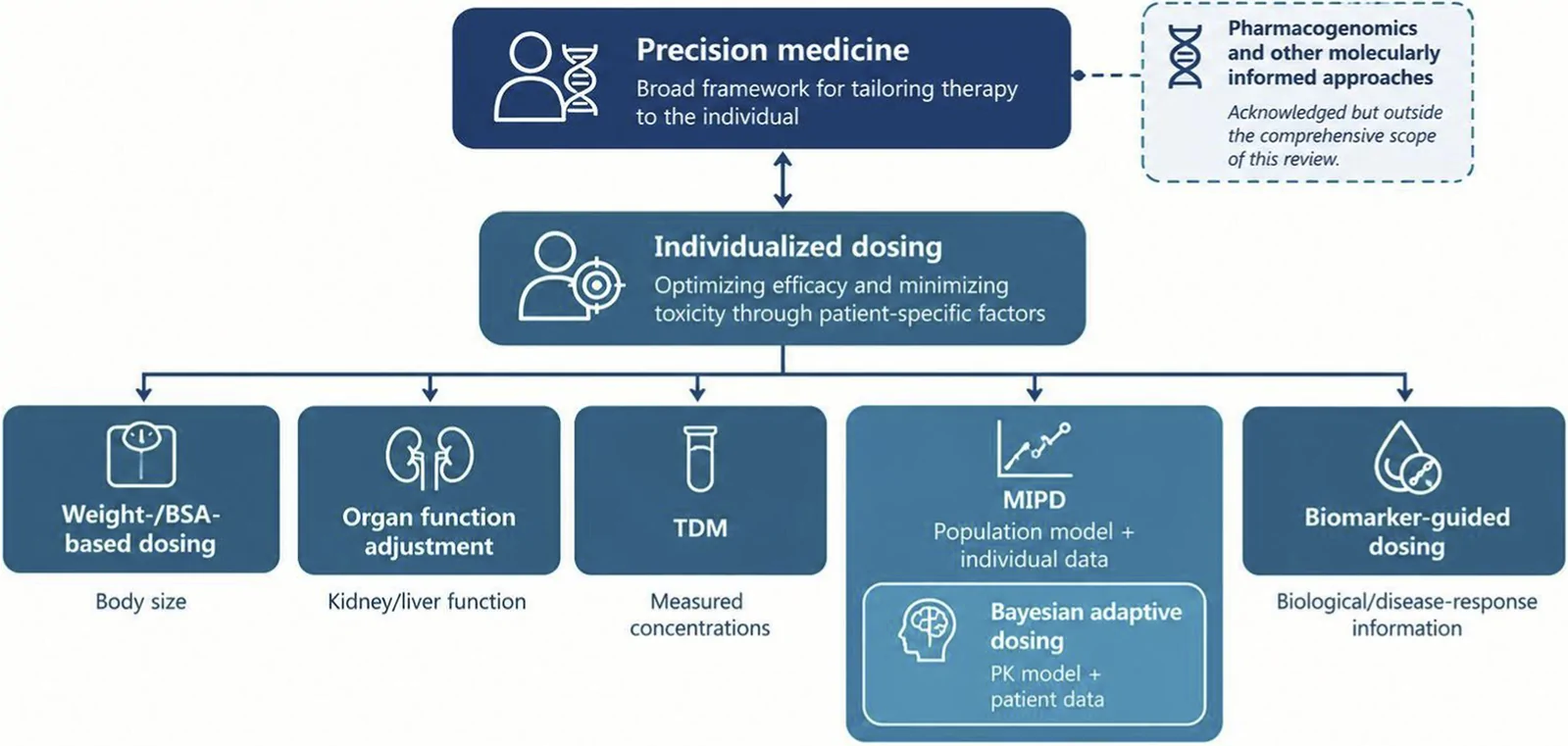
Conceptual framework illustrating individualized dosing as a component of precision medicine and the complementary approaches used to optimize drug therapy. BSA: body-surface area; MIPD: model-informed precision dosing; PK: pharmacokinetic; TDM: therapeutic drug monitoring.

This article was developed as a narrative review to provide a broad overview of individualized dosing strategies across major therapeutic classes. The literature was narratively synthesized according to the scope and objectives of the review, with emphasis on conventional and emerging approaches to dose individualization, pharmacokinetic considerations, therapeutic drug monitoring, model-informed precision dosing, and biomarker-guided dosing.

## 2 Emerging approaches for individualized dosing

Model-informed precision dosing (MIPD) is an advanced quantitative approach focusing on individualized dosing optimization by tailoring drug dosing to individual patient characteristics (Pérez-Blanco and Lanao, 2022; Minichmayr et al., 2024). MIPD extends beyond conventional therapeutic drug monitoring (TDM), which uses measured drug concentrations to maintain effectiveness while minimizing toxicity, by incorporating mathematical models to optimize dosing, while accounting for individual patient characteristics, drug concentration data, and multiple sources of variability. Consequently, robust model validation and qualification are essential before applying MIPD in clinical practice (Minichmayr et al., 2024). MIPD encompasses model-based approaches to dose optimization and may incorporate patient-specific covariates, drug concentration data, and other relevant sources of information. Bayesian adaptive dosing represents an important and widely implemented approach within MIPD.

Because traditional TDM is often underutilized due to challenges in coordinating sampling and interpreting results, Bayesian dosing represents a more advanced approach to individualized medicine (Donagher et al., 2017). Bayesian dosing is the most widely used and clinically implemented MIPD, first proposed in 1969 as an approach to accurately predict future drug doses tailored to individual patients (Sheiner, 1969). Bayesian dosing incorporates population pharmacokinetics (PKs) to individualize therapy, using models that identify and quantify sources of variability in drug concentrations across patient populations (Donagher et al., 2017). This information, often used to develop dosing nomograms, is leveraged to tailor drug doses to individual patients. Bayesian methods are effective because they enable us to “learn” the underlying reasons for an individual patient’s response, such as observed drug concentrations. They are particularly valuable when inter-patient variability in drug response cannot be fully explained by known covariates or patient factors such as body weight. Compared with approaches that are based on population methods alone, Bayesian methods are generally more accurate and can achieve target drug concentrations earlier in the course of therapy (Avent et al., 2013). Although the term “Bayesian” may imply a high degree of mathematical complexity, the underlying principles are, in fact, relatively straightforward. Figure 2 presents a flow diagram illustrating the process of Bayesian adaptive dosing.

**FIGURE 2:**
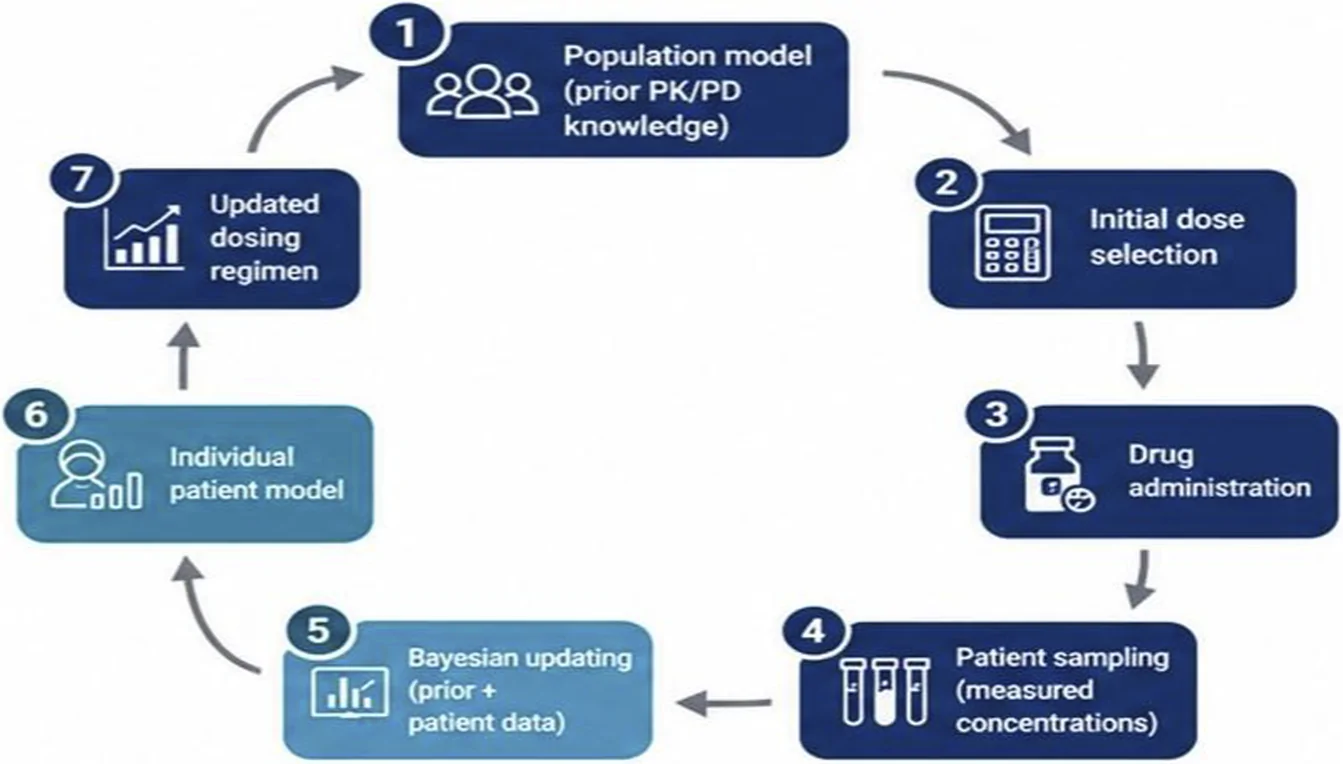
Bayesian adaptive dosing.

Despite its potential, clinical implementation of MIPD requires careful consideration of model validity and applicability. Many population PK models have limited external validation, and relatively few have been evaluated or implemented in new clinical settings (El Hassani and Marsot, 2023; El Hassani and Marsot, 2026). Model performance may also vary across populations, highlighting the importance of demographic and clinical similarity between model development and intended use populations (Taylor et al., 2023). For example, external validation of infliximab models in Korean patients with IBD demonstrated variable predictive performance across models and patient subgroups (Kim et al., 2025). Analytical differences may also affect MIPD performance; systematic differences between therapeutic drug-monitoring assays can bias measured concentrations and subsequent Bayesian dose estimation, as demonstrated for ustekinumab (Zhang et al., 2026). Thus, MIPD models should ideally be externally validated in representative populations using assays comparable to those used for model development.

Repurposing biomarkers as PD parameters to guide dosing is an attractive approach. Biomarker-guided dosing utilizes a measurable biological signals that can directly inform dose adjustment or provide information on disease activity and treatment response (Theile and Cho, 2019). Examples of biomarkers that directly guide dose adjustment include INR (international normalized ratio) for warfarin dosing, TSH (thyroid-stimulating hormone) for levothyroxine dosing, and ANC (absolute neutrophil count) for chemotherapy dose reductions or delays (Jonklaas et al., 2014; Freifeld et al., 2011). In contrast, biomarkers such as HbA1c (hemoglobin A1c), PSA (prostate-specific antigen), and CEA (carcinoembryonic antigen) are primarily used to assess treatment response or disease activity and may inform treatment intensification or regimen selection rather than directly determine dose adjustments (American Diabetes Association Professional Practice Committee for Diabetes, 2026; Gallus et al., 2000). Figure 3 illustrates the integration of biomarkers into dose individualization.

**FIGURE 3:**
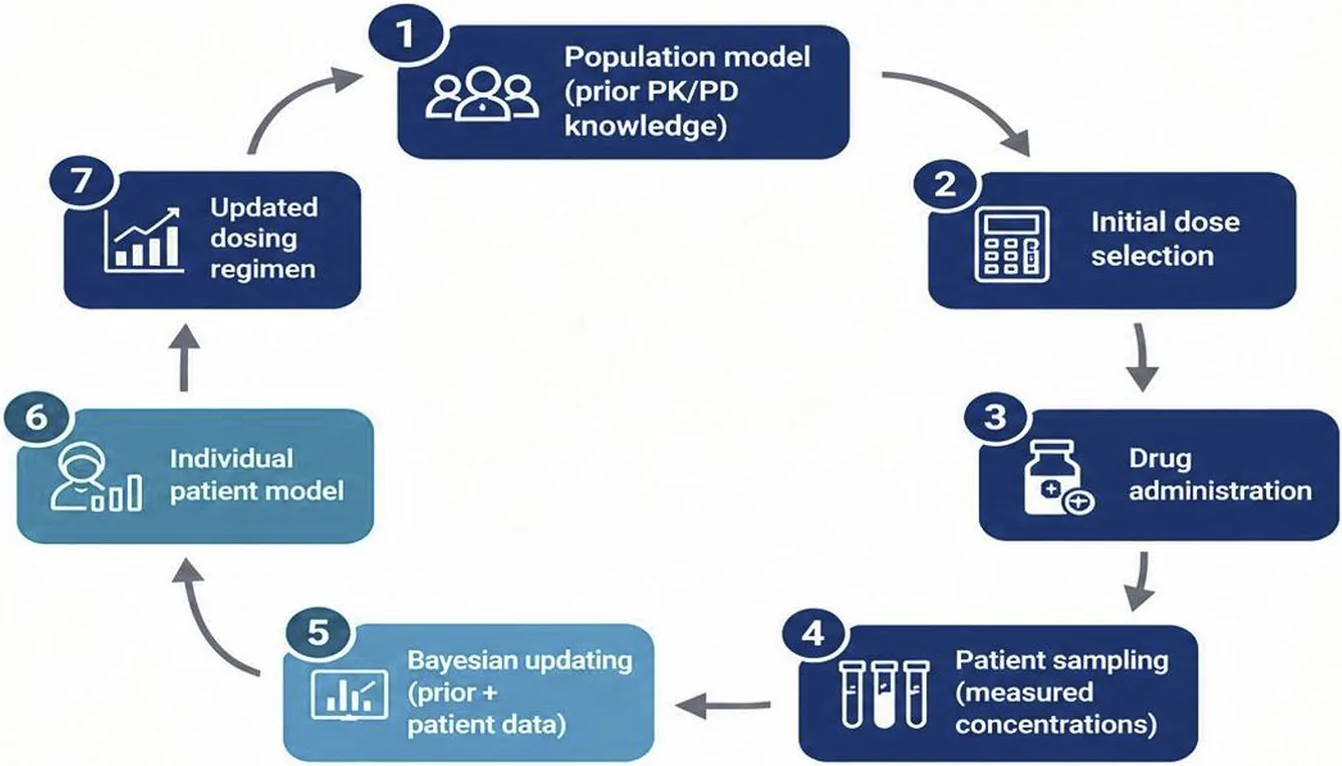
Biomarker-guided dosing.

## 3 Individualized dosing across therapeutic classes

### 3.1 Antimicrobials

The historical paradigm of antimicrobial administration has relied heavily on “flat” or fixed dosing strategies, applying a standardized regimen across diverse clinical populations. While logistically simple, this approach fundamentally fails to account for profound inter-individual and intra-individual PK and PD variability. Antimicrobials exhibit complex PK profiles driven by their physicochemical properties. Hydrophilic agents, including beta-lactams and glycopeptides, possess a Vd that mirrors extracellular fluid space, rendering them highly sensitive to physiological fluid shifts. Furthermore, the clearance of renally eliminated antimicrobials is drastically impacted by fluctuations in glomerular filtration rate. This variability becomes a critical safety and efficacy concern for antimicrobials with a narrow TI, where the margin between therapeutic success and concentration-dependent toxicity is exceedingly tight. Individualizing antimicrobial regimens based on patient-specific covariates is therefore paramount to maximizing effectiveness and minimizing toxicity in modern clinical practice (Cusumano et al., 2020).

To mitigate the inherent risks of flat dosing, TDM has traditionally been employed to guide therapy. Conventional TDM relies on measuring steady-state drug concentrations, most commonly trough levels, and utilizing linear pharmacokinetic equations for reactive dose adjustments. However, this approach is intrinsically limited by its retrospective nature and the assumption of steady-state kinetics, which is rarely maintained in acutely ill patients. Consequently, clinical pharmacy is experiencing a paradigm shift from traditional therapeutic drug monitoring (TDM) toward model-informed precision dosing (MIPD), in which Bayesian adaptive forecasting represents an important approach for individualizing drug dosing and estimating pharmacokinetic exposure (Wicha et al., 2021). Systematic reviews and meta-analyses confirm that the application of such advanced individualized dosing tools significantly reduces the risk of adverse events, such as nephrotoxicity, while substantially improving therapeutic target attainment (He et al., 2020).

The transition toward Bayesian forecasting and MIPD is most visibly demonstrated in the optimization of vancomycin and beta-lactam therapies. Historically, vancomycin guidelines recommended utilizing trough concentrations as a surrogate marker for the Area Under the Curve to Minimum Inhibitory Concentration (AUC/MIC) ratio. Mounting evidence, however, has demonstrated that trough-guided dosing frequently results in an unacceptably high incidence of acute kidney injury. Contemporary consensus guidelines now mandate a definitive shift toward direct AUC-guided dosing within a framework of MIPD, explicitly recommending Bayesian forecasting to achieve an optimal AUC/MIC target of 400–600 mgh/L (Matsumoto et al., 2022). Trial simulations comparing traditional PK equations to Bayesian-guided monitoring prove that Bayesian models provide superior predictive performance, achieve target AUCs more rapidly, and necessitate far less intensive blood sampling (Aljutayli et al., 2022).

Similarly, precision dosing for beta-lactam antibiotics is rapidly evolving. Beta-lactams rely on time-dependent bactericidal activity, necessitating that unbound drug concentrations remain above the MIC (ƒT > MIC) for a prolonged portion of the dosing interval. Standard intermittent infusions frequently fail to achieve these PD targets. Multicenter systematic evaluations emphasize the necessity of Bayesian forecasting for beta-lactams like piperacillin, where predicting individual clearance is vital to ensuring continuous target attainment without neurotoxic accumulation (Greppmair et al., 2023). Furthermore, recent clinical evaluations within critical care environments suggest that the integration of MIPD for beta-lactams not only optimizes pharmacokinetic parameters but also translates directly into improved clinical outcomes (Oakley et al., 2026).

Additionally, a recent systematic review and meta-analysis evaluating the clinical efficacy of individualized antimicrobials (including vancomycin, voriconazole, beta-lactams, and fluoroquinolones) found that individualized antimicrobial dosing improved target achievement, lowered treatment failure risk, and reduced nephrotoxicity, with no effect on length of stay or treatment duration. While dose individualization via TDM and MIPD maintains safe and effective drug levels, it faces implementation limitations, such as high costs and the requirement of significant resources (Sanz-Codina et al., 2023).

The implementation of MIPD is clinically essential for highly complex patient populations, where standard population models routinely fail to capture extreme physiological deviations. In the obese population, nonlinear alterations in both Vd, driven by excess adipose tissue, and altered renal clearance render traditional weight-based flat dosing wildly inaccurate. Dedicated MIPD frameworks and consensus guidelines are therefore required to prevent systemic underdosing or severe toxicity in patients with obesity (Märtson et al., 2025). Critically ill patients represent another distinct challenge, particularly those receiving continuous renal replacement therapy (CRRT). These patients may experience substantial and dynamic changes in drug clearance, with residual diuresis representing an important determinant of drug elimination. Consequently, individualized dosing supported by therapeutic drug monitoring and Bayesian forecasting may be particularly valuable for optimizing drug exposure in this population (Yu et al., 2023). Augmented renal clearance (ARC) represents another clinically important source of pharmacokinetic variability, particularly among critically ill patients. Enhanced renal elimination can substantially increase the clearance of renally eliminated antimicrobials, increasing the risk of subtherapeutic exposure with conventional dosing. A recent systematic review and meta-analysis found that ARC was significantly associated with failure to attain target antibiotic concentrations. This finding underscores the value of individualized, exposure-guided dosing strategies, including MIPD and therapeutic drug monitoring, particularly when substantial variability in renal clearance is anticipated (Alsalloum et al., 2025). Finally, pediatric populations require specialized approaches, as ontogeny and continuous developmental changes in organ function dramatically alter PK parameters. Fostering an understanding of this unique variability through age-calibrated population models is essential to attaining true precision medicine in pediatric care (Gijsen et al., 2021).

### 3.2 Chemotherapy

Traditionally, individualized-dosing strategies are widely employed with cytotoxic conventional chemotherapy, most commonly based on body surface area (BSA), with the core objective of minimizing interindividual variability in drug exposure and chemotherapy-related toxicities (Agema et al., 2025). Numerous chemotherapeutic agents demonstrate pharmacokinetic variability due to possessing narrow therapeutic indices. Treatment failure and severe toxicities, including myelosuppression, nausea, vomiting, and mucositis, can occur as a result of minor deviations in drug exposure (Smita et al., 2022).

Although BSA is correlated with physiological variables impacting drug disposition, including cardiac output, renal and hepatic function, and metabolic load, and despite its widespread utilization, it harbors important limitations. Several studies have shown that BSA only provides a modest fraction of interindividual pharmacokinetic variability (Thelwell et al., 2024). As a result, this prompted a major shift towards individualized dosing with particular conventional chemotherapies, including TDM, and model-informed precision dosing or Bayesian forecast approaches, which reflect a more precise PK and PD principles (Szpak et al., 2017; Goutelle et al., 2022). High-dose methotrexate (HD-MTX) is an antimetabolite chemotherapy with a narrow therapeutic window that requires therapeutic drug monitoring (TDM) to assess drug elimination and guide supportive care and leucovorin rescue. Although HD-MTX is generally dosed based on body surface area (BSA), its monitoring relies on serial plasma methotrexate concentrations at predefined time points, together with assessment of renal function and clinical status, to identify delayed elimination and guide management. This approach helps reduce the risk of HD-MTX-related toxicities, including myelosuppression, nephrotoxicity, hepatotoxicity, neurotoxicity, and gastrointestinal toxicity (Calabrò et al., 2025; Pourroy et al., 2025).

Carboplatin, a platinum-based chemotherapy, provides a distinct example of individualized chemotherapy dosing. Conventional carboplatin dosing commonly uses the Calvert formula, which estimates the dose required to achieve a target area under the concentration–time curve (AUC) based on renal function: dose (mg) = target AUC × (GFR + 25). Thus, the Calvert formula is an exposure-targeting dosing approach based on renal function rather than a Bayesian model-informed dosing strategy. Appropriate estimation of renal function remains an important consideration, particularly in patients with obesity or altered renal function. The Calvert formula allows targeting of a specific AUC, thereby improving consistency of drug exposure and potentially reducing variability in response and toxicity (Leiva and Yamauchi, 2023; Schwenk, 2024).

In contrast, Bayesian model-informed dosing combines population pharmacokinetic models with patient-specific observations, such as measured drug concentrations, to estimate individual pharmacokinetic parameters and refine subsequent dosing. Bayesian forecasting through TDM is utilized with the alkylating agent busulfan, particularly in pediatric patients. Given its narrow therapeutic index, busulfan has a specific target AUC profile, with underexposure potentially increasing the risk of disease relapse or graft rejection in conditioning protocols for allogeneic stem cell transplantation, whereas overexposure may increase the risk of post-transplant complications, including veno-occlusive disease (VOD), a major cause of non-relapse mortality (Bazbaz et al., 2025; Alomari et al., 2025). On the contrary, particular conventional chemotherapeutic agents have gradually moved towards flat dosing strategies, especially when pharmacokinetic variabilities are less pronounced. For instance, oral hypomethylating agents, including azacitidine and decitabine, are given as fixed doses of 300 and 35 mg, respectively. Clinical trials have demonstrated that flat dosing achieves the target concentration while minimizing related toxicities. Additionally, in the QUAZAR phase 3 clinical trial, oral fixed-dose azacitidine was associated with a longer overall survival and relapse-free survival as maintenance therapy in intermediate and high-risk acute myeloid leukemia, thereby maintaining efficacy across different weight variations (Wei et al., 2023; Garcia-Manero et al., 2024).

### 3.3 Biologics and immunotherapy

The era of biologics and immunotherapies has significantly impacted traditional dosing principles. Compared to conventional chemotherapy, numerous monoclonal antibodies and immunotherapies exhibit consistent pharmacokinetic and pharmacodynamic profiles, enabling flat dosing for certain agents and specific indications (Papachristos et al., 2023).

Flat or fixed dosing of biologics and immunotherapies is supported by particular characteristics, including minimal tissue distribution as a result of the large size and hydrophilic nature of these agents, which correlates with a wider therapeutic window, leading to lower interindividual pharmacokinetic variability (Fan et al., 2026). Although weight-based dosing is still recommended for certain biologics and immunotherapies, flat dosing has become increasingly adopted due to shorter infusion or administration time, lower risk of dosing errors, operational convenience, and reduced overall costs (Hendrikx et al., 2017).

Body composition has relatively less of an impact on drug exposure of monoclonal antibodies, which are mainly metabolized and eliminated through target-mediated drug disposition and intracellular catabolism rather than hepatic metabolism or renal elimination (Fan et al., 2026). Flat dosing generally yields a comparable drug exposure to weight-based dosing based on population-based pharmacokinetic studies. For instance, trastuzumab is a monoclonal antibody that specifically targets human epidermal growth factor receptor 2 (HER2), which has traditionally been administered intravenously through weight-based dosing in breast cancer. It is now FDA-approved as a subcutaneous injection with a flat dose of 600 mg given every 3 weeks after demonstrating consistent drug concentrations regardless of weight variations through population-based pharmacokinetic analyses (U.S. Food and Drug Administration, 2026).

In the phase 3 non-inferiority HannaH trial, 596 patients with HER2-positive stage I-III breast cancer were randomized to receive either weight-based intravenous trastuzumab given as an 8 mg/kg loading dose followed by 6 mg/kg every 3 weeks or flat dose subcutaneous trastuzumab as 600 mg every 3 weeks. The study found that pre-surgical trough levels were higher with subcutaneous than with intravenous administration (69.0 vs. 51.8 μg/mL). Clinically, pathological complete response was comparable between the two formulations (45.4% versus 40.7%), and the 6-year event-free survival and overall survival rates were also similar for flat dose and weight-based dosing (Jackisch et al., 2019). However, these findings should be interpreted cautiously, as the comparison involved different formulations and routes of administration in addition to different dosing strategies; therefore, differences in exposure cannot be attributed solely to fixed-dose versus weight-based dosing. Moreover, real-world studies have shown that early drug exposure resulted in variability with flat dosing. In one cohort study that included 50 patients, trastuzumab target concentrations were statistically significantly lower with flat dose subcutaneous dosing compared to weight-based intravenous administration during the first cycle (67.6% vs. 93.8%; P = 0.042) (González García et al., 2020). However, these real-world studies included small cohorts and require larger-scale studies to influence current dosing recommendations.

Over the last decade, flat dosing has become more prominent with immune checkpoint inhibitors (ICIs). Agents such as nivolumab and pembrolizumab were historically administered using weight-based dosing; however, numerous pharmacokinetic population analyses have proven that weight was responsible for a small proportion of interpatient variability in certain malignancies. For instance, nivolumab has shifted from 3 mg/kg every 2 weeks to 240 mg every 2 weeks or 480 mg every 4 weeks, whereas pembrolizumab was administered as 2 mg/kg every 3 weeks to a fixed dose of 200 mg every 3 weeks (Hendrikx et al., 2017). Although flat dosing has been shown to maintain efficacy across certain tumor types, toxicity outcomes, particularly in patients with extreme body weights, remain a concern. In a real-world retrospective study that included 313 underweight patients (<50 kg), weight-based dosing was associated with lower immune-mediated toxicities, including hypothyroidism and colitis, compared with flat dosing (21.3% vs. 32.1%) as well as lower hospitalization rates associated with these toxicities (13.1% vs. 21.8%) (Hamad et al., 2024).

Therapeutic drug monitoring (TDM) represents an established approach to individualizing anti-TNF therapy, particularly for infliximab and adalimumab. Measurement of trough drug concentrations, with assessment of anti-drug antibodies when appropriate, can help identify pharmacokinetic causes of primary non-response or secondary loss of response and guide dose intensification or treatment switching. Although reactive TDM is widely used, the role of proactive TDM remains less certain, with recent guidance supporting its use selectively, particularly during maintenance treatment with intravenous infliximab, while evidence for routine proactive TDM with adalimumab and other biologics remains limited (Kawano-Dourado et al., 2024).

### 3.4 Anesthetics and critical care agents

Many anesthetic and critical care drugs pose a particular challenge for individualized dosing because of their narrow therapeutic indices, high lipophilicity, and the profound pharmacokinetic alterations associated with critical illness (Devlin et al., 2018; Erstad and Barletta, 2020). Unlike many chronic therapies, these medications require rapid titration to achieve the desired therapeutic effect while minimizing potentially life-threatening adverse events (Devlin et al., 2018; Roberts and Lipman, 2009). Consequently, selecting an appropriate body-size dosing scalar, such as total body weight (TBW), ideal body weight (IBW), lean body weight (LBW), or fat-free mass (FFM), is a cornerstone of dose optimization. Furthermore, the pharmacokinetics of many anesthetic and critical care agents are profoundly influenced by obesity, critical illness, extracorporeal support (e.g., extracorporeal membrane oxygenation and continuous renal replacement therapy), and augmented or impaired drug clearance, making standardized dosing strategies unreliable in many patients (Erstad and Barletta, 2020; Alsalloum et al., 2025). Accordingly, individualized dosing in perioperative and critical care settings aims not only to optimize drug exposure but also to facilitate rapid achievement of therapeutic targets, minimize hemodynamic instability, avoid prolonged drug effects, and promote timely recovery from anesthesia and sedation.

According to Johnson et al., propofol is a highly lipophilic drug whose distribution into adipose tissue makes total body weight (TBW)-based dosage problematic in obese individuals (Johnson et al., 2018). In 54 obese patients (mean BMI ∼ 49 kg/m^2^) undergoing sleeve gastrectomy, Yıldırım and colleagues found that TBW-based induction significantly increased hemodynamic instability, including reductions in systolic blood pressure, mean arterial pressure, and heart rate, and bispectral index values below 40, indicating excessive anesthetic depth, compared to lean body weight (LBW)-based dosing (p < 0.05). Neither group showed any intraoperative awareness (Yıldırım et al., 2024). In addition, Djalalimotlagh et al. showed in a double-blind RCT that in morbidly obese individuals, fat-free mass (FFM)-based induction produced better anesthetic depth than IBW-based dosage without hemodynamic differences between groups (Djalalimotlagh et al., 2023). Additionally, Xu and colleagues noted that TBW-based dosage was linked to enhanced brain sensitivity and overdose risk and determined that 2.31–3.57 mg/kg LBW was the optimum induction dose range in morbidly obese individuals (Xu et al., 2020). Although patients with BMI ≥40 kg/m^2^ were less likely to achieve sedation targets with ABW-based dosing, IBW-based propofol infusion was demonstrated to be as safe and effective as ABW-based dosing in the ICU setting, supporting weight-adjusted strategies in severe obesity (Gimelbrand et al., 2022; Hayes and Esteves, 2023). Kroeger et al. found a substantial difference between weight-based ideal propofol doses and actual doses given during IVF oocyte retrieval in 1,564 women (mean difference: 196.83 mg; p ≤ 0.001), which was linked to clinically significant drops in blood pressure and heart rate across all cohorts, further highlighting the clinical significance of dosing discrepancies (Kroeger et al., 2025). Beyond conventional weight-based dosing, pharmacokinetic modeling approaches have been developed to account for age-related pharmacokinetic variability and inform propofol administration. In neonates and infants, pharmacokinetic modeling has been used to evaluate existing infusion regimens and derive age-specific manual infusion regimens designed to achieve target propofol concentrations, illustrating the potential of model-informed approaches to optimize dosing in pediatric populations (Morse et al., 2019).

Ketamine provides more dosage flexibility due to its broader TI. A fixed intramuscular dose of 250 mg was found to be effective in 80% of prehospital patients with hyperactive delirium and severe agitation. There was no significant correlation between weight-based dose and intubation risk, which supports fixed dosing as a practical option in time-sensitive emergency settings where weight assessment is impractical, though not as a general principle (O’Brien et al., 2024).

While a fixed dose of bupivacaine may provide adequate and safe anesthesia in patients with a normal body weight (McAllister and Patel, 2026), Siddiqui et al. demonstrated that individualized dosing based on both weight and height is superior for patients undergoing elective cesarean delivery compared with dosing based on height alone. This approach was associated with a lower incidence and severity of hypotension, as well as reduced ephedrine requirements (Siddiqui et al., 2016). These benefits appear to be particularly pronounced in patients with obesity (McCulloch and Littlewood, 1986).

In terms of neuromuscular blocking agents (NMBAs), non-depolarizing agents like rocuronium should be dosed on IBW or LBW to prevent prolonged blockade in obese patients, while succinylcholine should be dosed based on TBW due to the proportionate increase in pseudocholinesterase activity with body mass (Erstad and Barletta, 2021). In terms of reversal, Jung and colleagues found no significant difference between a fixed dose of 200 mg and weight-based sugammadex (2 mg/kg) in patients within normal weight ranges (2.3 vs. 2.0 min to train-of-four recovery, p = 0.52); however, fixed dosing carries the risk of insufficient reversal in heavier patients, upholding the justification for weight-based individualization across larger populations (Jung et al., 2025).

The key principles, dosing strategies, and clinical considerations underlying individualized dosing across the major therapeutic classes discussed in this review are summarized in Table 1.

**TABLE 1:** Comparative overview of individualized dosing across selected therapeutic classes.

Aspect	Antimicrobial agents	Chemotherapy	Monoclonal antibodies and immunotherapy	Anesthetics and critical care agents
Primary rationale for individualized dosing	Marked PK/PD variability, altered Vd and clearance, narrow therapeutic index	Narrow therapeutic index and interindividual PK variability	Drug-specific PK characteristics; individualized dosing for selected agents, whereas flat dosing is appropriate for many monoclonal antibodies	Narrow therapeutic index (many agents), lipophilicity, altered PK in obesity and critical illness
Major patient-specific factors	Body weight, renal function, critical illness, obesity, CRRT, pediatrics	BSA, renal function (e.g., carboplatin), obesity, age	Body weight for selected agents; body weight has limited influence on PK variability for many monoclonal antibodies	Body weight, body composition (LBW, FFM, IBW), obesity, critical illness
Predominant dosing strategy	Weight-based dosing with TDM/MIPD	BSA-based dosing with drug-specific individualization	Primarily flat dosing; weight-based dosing for selected biologics	Weight-based dosing using appropriate body-size scalar; fixed dosing for selected agents
Role of therapeutic drug monitoring (TDM)	Established standard for several agents (e.g., vancomycin, voriconazole)	Essential for selected drugs (e.g., high-dose methotrexate, busulfan)	Established for selected anti-TNF agents (particularly infliximab and adalimumab); reactive TDM is more widely supported than routine proactive TDM	Limited role; clinical titration predominates
Role of Bayesian/MIPD	Well-established and increasingly recommended	Emerging for selected agents (e.g., busulfan, carboplatin)	Limited current application	Limited but potential future application
Common consequences of non-individualized dosing	Treatment failure, nephrotoxicity, neurotoxicity, subtherapeutic exposure	Treatment failure, myelosuppression, organ toxicity	Increased toxicity or unnecessary drug exposure in selected populations	Hemodynamic instability, excessive anesthetic depth, prolonged drug effects, inadequate reversal
Representative individualized agents	Vancomycin, β-lactams, aminoglycosides, voriconazole	Methotrexate, carboplatin, busulfan	Infliximab, adalimumab, rituximab, bevacizumab, intravenous trastuzumab	Propofol, rocuronium, succinylcholine, sugammadex
Examples where flat dosing is appropriate	Rare	Selected oral hypomethylating agents	Many monoclonal antibodies (e.g., pembrolizumab, nivolumab, subcutaneous trastuzumab)	Selected agents (e.g., intramuscular ketamine in emergency settings; sugammadex and bupivacaine in selected normal-weight patients)

Anti-TNF, agents, anti-tumor necrosis factor agents; BSA, body surface area; FFM, fat-free mass; IBW, ideal body weight; LBW, lean body weight; MIPD, model-informed precision dosing; PK/PD, pharmacokinetic/pharmacodynamic; TDM, therapeutic drug monitoring; Vd, volume of distribution.

Importantly, improved pharmacokinetic exposure or attainment of predefined PK/PD targets should not be considered synonymous with improved patient-centered outcomes. Although individualized antimicrobial dosing strategies, including TDM and MIPD, can improve target attainment and may reduce adverse events such as nephrotoxicity and treatment failure, these benefits do not consistently extend to broader clinical outcomes. For example, evidence has not consistently demonstrated improvements in clinical response, mortality, or length of hospital stay despite improved target attainment. Therefore, PK/PD target attainment should be regarded as an important intermediate measure of dosing quality rather than as a surrogate for clinical benefit in all settings (He et al., 2020; Sanz-Codina et al., 2023).

## 4 Challenges of individualized/weight-based dosing

Beyond the intrinsic complexity of dose preparation and the practical limitations related to dosage-form availability, individualized dosing strategies introduce several operational and clinical challenges. Weight-based (mg/kg) and body surface area–based (mg/m^2^) regimens frequently require dose calculations that do not correspond to commercially available vial sizes or tablet strengths (Pan et al., 2016; Pai, 2012; Hendrikx et al., 2017). This may necessitate dose rounding, tablet splitting, reconstitution of parenteral formulations, partial vial use, or compounding of extemporaneous preparations (Pan et al., 2016; Pai, 2012). Each additional manipulation step increases preparation time, introduces variability in dose accuracy, and may compromise sterility or stability in the case of parenteral agents (Hendrikx et al., 2017; Ansel and Stockton, 2017). In institutional settings, such requirements may also increase pharmacy workload and drug wastage, particularly when single-use vials are involved (Hendrikx et al., 2017; Bach et al., 2016).

Additionally, calculation errors represent a substantial challenge in individualized dosing strategies. These regimens require multiple sequential steps, accurate measurement of anthropometric parameters, unit conversions, formula application, dose rounding, and preparation of the final formulation (Keers et al., 2013). Each step introduces potential sources of error, including transcription mistakes, incorrect unit conversion (e.g., pounds to kilograms), misapplication of BSA formulas, decimal point errors, and inappropriate rounding (Keers et al., 2013; Aronson, 2009). In high-risk medications, particularly those with a narrow TI, such errors may result in clinically significant underexposure or toxicity (Derendorf and Meibohm, 1999; Aronson, 2009).

The cognitive burden associated with manual calculations further increases error susceptibility, particularly in high-acuity or time-constrained clinical environments (Keers et al., 2013). Although computerized provider order entry (CPOE) systems and clinical decision support tools reduce mathematical errors, they remain dependent on accurate data entry (Slight et al., 2017). Erroneous input of weight, height, renal function, or laboratory parameters can propagate through automated systems and generate systematically incorrect doses (Slight et al., 2017).

Moreover, the availability and reliability of anthropometric measurements may present practical obstacles. In critically ill, immobilized, or emergency department patients, obtaining an accurate body weight can be challenging, leading to reliance on estimated values that may deviate substantially from true measurements, especially in patients at extremes of body habitus (Bloomfield et al., 2006). Height measurement, required for BSA-based dosing, may be unavailable or inaccurate in bedridden individuals (Bloomfield et al., 2006). Fluid overload, edema, ascites, or rapid perioperative weight fluctuations can further distort weight-based calculations by misrepresenting pharmacokinetically relevant compartments such as lean body mass (Pai and Bearden, 2007). Furthermore, interprofessional communication gaps, such as discrepancies between outpatient and inpatient recorded weights, can result in inconsistent dosing across care transitions. In pediatric and geriatric populations, age-related physiological changes further complicate the interpretation of weight-based dosing algorithms (Kearns et al., 2003).

Collectively, while individualized dosing is intended to optimize pharmacokinetic precision and improve therapeutic outcomes, it introduces logistical, operational, and systems-level complexities that may themselves contribute to variability, inefficiency, and medication error in real-world clinical practice (Keers et al., 2013; Slight et al., 2017).

## 5 Conclusion

Individualized dosing remains a cornerstone of precision medicine by optimizing therapeutic efficacy while minimizing toxicity through patient-specific dose selection. As demonstrated across antimicrobial agents, chemotherapy, monoclonal antibodies and immunotherapies, and anesthetic and critical care drugs, no single dosing strategy is universally applicable. Instead, the optimal approach should be guided by the pharmacokinetic and pharmacodynamic properties of the drug, the degree of interindividual variability, and patient-specific characteristics. While conventional approaches, including weight- and body surface area–based dosing, continue to play a pivotal role for many medications, fixed dosing is appropriate for selected agents with predictable pharmacokinetics. Despite the logistical and operational challenges associated with individualized dosing, ongoing advances in pharmacokinetic modeling, therapeutic drug monitoring, and clinical decision support tools are expected to improve dosing precision and facilitate implementation in routine clinical practice. Continued research should focus on refining individualized dosing strategies and generating high-quality evidence to support their application across diverse patient populations.
